# NSII: a novel soybean identification index based on Sentinel-2 imagery for accurate and efficient soybean mapping

**DOI:** 10.3389/fpls.2026.1788686

**Published:** 2026-04-01

**Authors:** Xiufang Zhu, Yinhui Wang, Anzhou Zhao, Dan Li

**Affiliations:** 1 State Key Laboratory of Remote Sensing and Digital Earth, Beijing Normal University, Beijing, China; 2 Key Laboratory of Environmental Change and Natural Disaster, Ministry of Education, Beijing Normal University, Beijing, China; 3 School of Earth Science and Engineering, Hebei University of Engineering, Handan, China; 4 School of Mining and Geomatics Engineering, Hebei University of Engineering, Handan, China

**Keywords:** class separability, crop mapping, Sentinel-2 data, soybean identification, spectral separability

## Abstract

Accurate mapping of soybean cultivation areas is crucial for agricultural monitoring, resource management, and food security. However, the spectral overlap between soybean and other crops, such as corn, poses significant challenges for remote sensing-based identification. This study proposes a novel soybean identification index (NSII), which is calculated using the second red-edge band (RE2), the first short-wave infrared band (SWIR1), and the Enhanced Vegetation Index (EVI) derived from Sentinel-2 imagery within the optimal time window identified through spectral feature analysis. NSII was implemented in 12 major soybean producing regions in the United States and China over a three-year period (2020-2022). Experimental results from 2020 to 2022 show that the average accuracy of NSII is 0.85, and the average F1 score is 0.80. Compared with the existing Soybean Mapping Composite Index (SMCI), the accuracy increased by 8 percentage points and the F1 score increased by 6 percentage points. NSII also exhibits strong stability and transferability, with consistent performance across diverse climatic and cropping conditions. This study provides a robust and efficient tool for soybean mapping, offering significant potential for precision agriculture and sustainable resource management.

## 1 Introduction

Accurate spatial distribution maps of crops are important for monitoring and evaluating farmland conditions, precision agriculture management, agricultural disaster response and agricultural policy formulation (Wu et al., 2023). It is usually time-consuming and laborious to utilize traditional agricultural survey methods to classify and map crops, while remote sensing technology, with its ability to acquire data macroscopically and rapidly, has become an important means of crop mapping (Gao and Zhang, 2021), which greatly saves the costs of manpower, material, financial and time, and effectively makes up for the shortcomings of traditional survey methods (Choukri et al., 2024). In recent years, the application of high-resolution (temporal and spatial) remote sensing data in crop identification has become more and more widespread (Xu et al., 2021).

Crop spectral characteristics exhibit distinct variations across species. (Farmonov et al., 2023; Sidike et al., 2019; She et al., 2020), constituting the most vital features for crop type identification (Agilandeeswari et al., 2022). The spectral differences in remote sensing between different crops stem from their anatomical structures (such as the arrangement of cells in monocotyledons and dicotyledons, leaf morphology, and surface characteristics), biochemical components (absorption or scattering of specific wavelengths of light by photosynthetic pigments, water, cellulose, etc.), phenological stages (changes in growth status at different growth stages), and growth environment (indirect effects of water, soil, light, etc. on leaf characteristics). The remote sensing spectral differences of major crops (rice, corn, wheat, soybeans) are primarily reflected in the chlorophyll absorption intensity in the visible light band, leaf structure scattering ability in the near-infrared band, red edge band position, and water absorption peak in the short-wave infrared band. Monocots (rice, corn, wheat) display markedly higher near-infrared reflectance and red-edge shifts compared to dicotyledonous soybeans, attributable to their distinct leaf architectures and chlorophyll distributions. Rice’s characteristically high moisture content produces pronounced short-wave infrared absorption peaks distinguishing it from xerophytic crops, whereas corn’s unique foliar structure generates diagnostic near-infrared and green-band reflectance signatures differentiating it from both wheat and soybeans.

Vegetation indices obtained by mathematical operations based on multiple bands of remotely sensed imagery can achieve the effect of enhancing the spectral characteristics of vegetation and are often used for crop type identification and distribution mapping studies (de Oliveira Maia et al., 2023; Yang et al., 2022). For example, Huang et al. (2022) used Normalized Difference Vegetation Index (NDVI) and Normalized Difference Water Index (NDWI) as inputs to the random forest model to map winter wheat in Henan Province, China, in 2020 at 10 m resolution. Qu et al. (2021) used NDVI time series data to derive four key growth nodes of winter wheat to construct a decision tree to realize the extraction of winter wheat. Liu et al. (2020) successfully identified rice using differences in Enhanced Vegetation Index (EVI) and Yellowness Index (YI) at different times of the year.

Some researchers also focus on developing spectral indices for the identification of specific crop types to enhance their specific spectral features and improve their identification accuracy. For example, Shi and Huang (2015) developed a Normalized Weighted Difference Water Index (NWDWI) to identify rice, and used this index to monitor the planted area of single-season and double-season early and late rice in the Yangtze River Delta region, and verified the accuracy of the method using agricultural census data. Tao et al. (2023) proposed a novel Phenology-based Winter Rapeseed Index (PWRI) and utilized the index to map the distribution of winter oilseed rape in the Yangtze River Basin. PWRI was shown to have good separability between winter oilseed rape and other crops throughout the flowering period, and the method was applied to the middle reaches of the Yangtze River, with an overall accuracy and kappa coefficient of more than 92% and 0.85%, respectively. Wang et al. (2021) developed a White Bolls Index (WBI) based on the unique canopy characteristics of cotton during the fluffing stage and applied it to cotton identification in Missouri’s 8th District, California’s 21st District, Georgia’s 8th District, and Shihezi and its surrounding areas in China’s North Xinjiang, with an overall accuracy rate of over 82%. Ashourloo et al. (2020) developed a new spectral feature for potato mapping by analyzing the spectral features of potato. They calculated the sum of the differences in reflectance between the red and near-infrared (NIR) bands from planting to the peak greenness stage, along with the ratio of NIR to red reflectance at peak greenness and the NIR reflectance at harvest. This approach effectively distinguished potatoes from other crops.

Soybean is the main raw material for high-protein food, livestock feed and edible oil, and has an important position in world food production (Li et al., 2021). Accurate identification of soybean cultivation areas is of great significance in guaranteeing food security (Zhu et al., 2022). Mapping and monitoring of the spatial distribution of soybean (Li et al., 2019) provides an important basis for agricultural decision-making, and farmers and agricultural decision-makers can adjust their planting plans based on these data to optimize the layout of soybean planting and resource allocation, so as to achieve the optimal benefits of soybean production. For this reason, some researchers have also focused on the development of soybean remote sensing identification index.

Soybean and corn are grown almost simultaneously in many regions and their spectra are highly similar in most phenological stages, making them easily confused (Bandaru et al., 2020). Therefore, when developing soybean identification indices, researchers tend to focus on the differences between soybean and corn spectral characteristics at specific phenological stages (Zhong et al., 2016). For example, Chen et al. (2023) proposed the Greenness and Water Content Composite Index (GWCCI) for soybean mapping by utilizing the differences between soybean and other crops in shortwave infrared (SWIR) bands and NDVI. Xiao et al. (2024) developed a Soybean Mapping Composite Index (SMCI) based on the optimal time window using Sentinel-2 imagery, which coupled three red-edge bands (RE2, RE3, and RE4), the near-infrared (NIR) band, the SWIR band, the EVI, and the Green Chlorophyll Vegetation Index (GCVI). The GWCCI only uses the SWIR band and the NDVI, neglecting the role of the red-edge band in soybean identification, and the NDVI index is prone to overfitting in areas of lush vegetation. The SMCI couples multiple features, which increases the computational complexity and the amount of data processing, and at the same time, highly correlated features may lead to feature redundancy problems.

In this study, we propose a novel remote sensing index for soybean identification, aiming to enhance the accuracy of soybean distribution mapping. Addressing the challenge of spectral similarity between soybeans and corn across multiple growth stages, we integrated visible, near-infrared, red edge, and shortwave infrared features to maximize information utilization across different bands while minimizing feature redundancy. Through validation across diverse cropping patterns and field characteristics in different regions and years, this study not only demonstrates the new index’s effectiveness in soybean identification but also compares it with the existing Soybean Mapping Composite Index (SMCI). This study can enrich the selection of features for soybean mapping and help accurate soybean distribution mapping research.

## 2 Materials

### 2.1 Study sites

**Figure 1:**
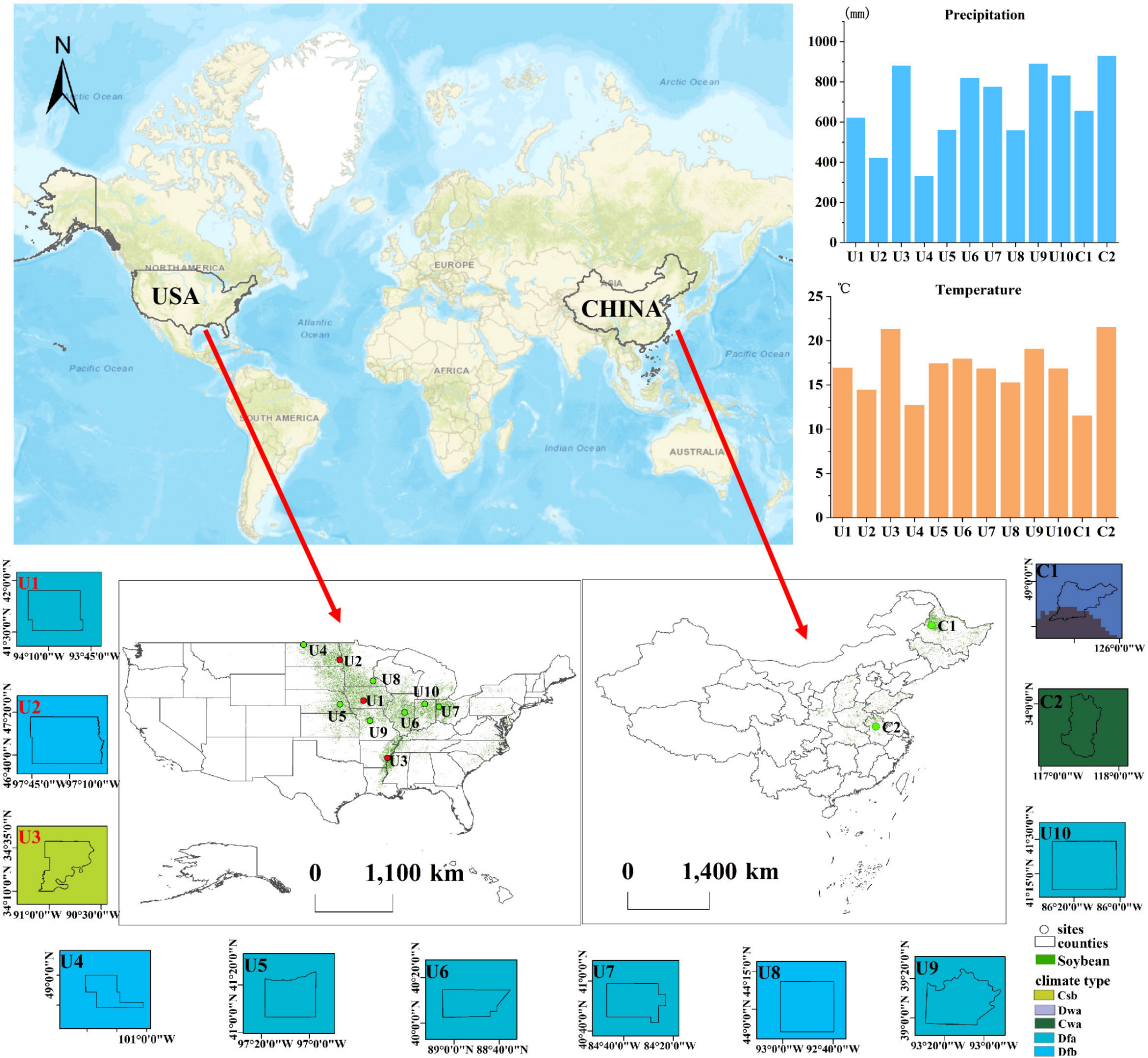
Spatial distribution, climate, average temperature, and total precipitation in 10 US counties (See **Table 1** for details).

As one of the largest soybean producers in the world, the U.S. accounts for about 23.9% of the global soybean acreage in 2023 (Volkova and Smolyaninova, 2024). As the world’s largest soybean consumer and importer, changes in China’s soybean cultivation have a significant impact on the global soybean supply and demand balance, market prices, and food security. In 2017, China’s soybean production accounted for approximately 3.57% of the global total (Di et al., 2023). In view of the different soybean planting conditions in China and the United States, this study selected 12 representative areas in the main soybean producing areas of the United States and China, which differed in terms of climate type, rainfall, temperature, and cropping structure (**Figure 1**; **Table 1**). Of these, the first three regions (U1-U3) were used for the development of the novel soybean identification index (NSII), and the remaining regions (U4-U10, C1-C2) were used for testing NSII. U1-U3 span three distinct Köppen climate types, illustrating the diversity of climates within the study area. The Köppen climate classification system, developed by Beck et al. (2018), categorizes global climates based on thresholds and seasonality of monthly air temperature and precipitation. The study area selected in this study contains five Köppen climate types, namely Mediterranean climate (warm summer) (Csb), Humid continental climate (dry winter, hot summer) (Dwa), Humid subtropical climate (dry winter, hot summer) (Cwa), Humid continental climate (no dry season, hot summer) (Dfa), and Humid continental climate (no dry season, warm summer) (Dfb).

**Table 1:** Detailed description of the study area.

Sites	Location	Main crops	Soybean		
			Planting ratio (%)	Sowing time	Harvest time
U1	Dallas, Iowa	Soybean, corn	46.86	late Apr-early May	late Sep-middle Nov
U2	Cass, North Dakota	Soybean, corn, spring wheat	46.82	late Apr-early May	late Sep-early Oct
U3	Phillips, Arkansas	Soybean, corn, cotton, rice	54.29	early Apr-early May	early Sep-late Oct
U4	Renville, North Dakota	Soybean, corn, spring wheat	35.95	late Apr-early May	late Sep-early Oct
U5	Butler, Nebraska	Soybean, sorghum, corn, winter wheat	41.55	late Apr	late Sep-early Nov
U6	De-witt, Illinois	Soybean, corn, winter wheat	50.17	late Apr	late Sep-early Nov
U7	Van-wert, Ohio	Soybean, corn, winter wheat	56.73	late Apr	late Sep-early Nov
U8	Dodge, Minnesota	Soybean, sunflower, corn, spring wheat	40.41	middle Apr	middle Sep-late Nov
U9	Saline, Missouri	Soybean, corn, cotton, rice	48.74	late Apr	late Sep-late Nov
U10	Marshall, Indiana	Soybean, corn, winter wheat	38.17	late Apr	middle Sep-late Nov
C1	Nehe	Soybean, corn, rice, potato	45.8%	middle May	late Sep-late Oct
	Heilongjiang				
C2	Lingbi	Soybean, corn, wheat	22.1%	late May	late Sep-late Oct
	Anhui				

**Figure 2:**
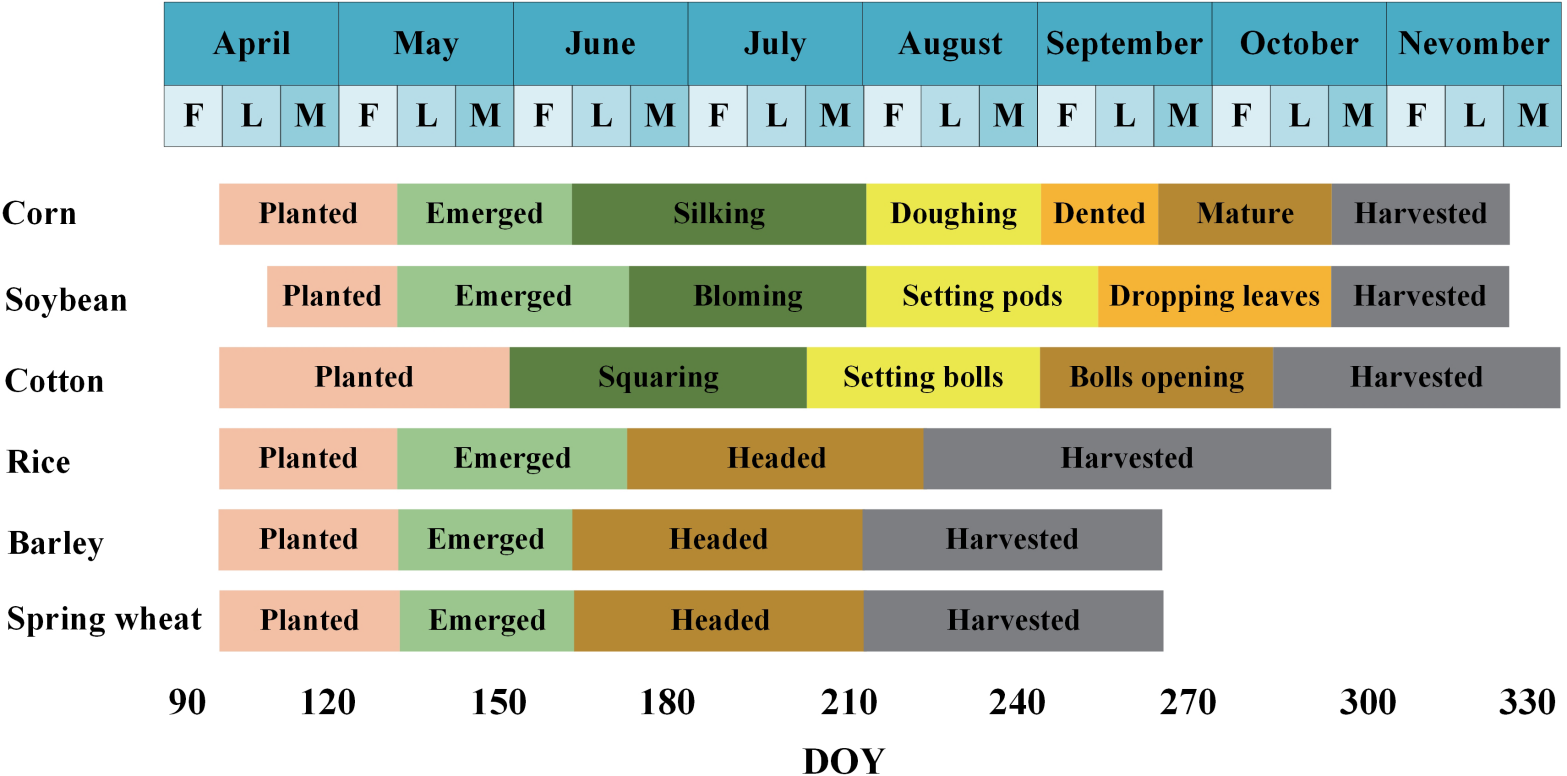
Phenological calendar of major crops in study areas U1-U3.

The first region (U1), located in Dallas County, Iowa. Iowa is one of the major soybean producing regions in the United States and has one of the highest soybean production in the nation (Hosseini et al., 2020). Dallas County has a Humid continental climate (no dry season, hot summer) (Dfa). Rainfall and high temperatures are concentrated in the summer months. The major crops grown in the county are soybean and corn. The second region (U2), located in Cass County, North Dakota, has a humid continental climate (no dry season, warm summer) (Dfb) with cold winters, and moderate but unevenly distributed annual precipitation. The broad plains topography of North Dakota provides unique conditions for agricultural development (Hanson et al., 2022). The major crops grown in Cass County are soybean, corn, and spring wheat. The third region (U3) is located in Phillips County, Arkansas. Before 2005, the planting industry of Arkansas was dominated by cotton, but the proportion of soybean and rice cultivation gradually increased after 2005. Arkansas belongs to the mediterranean climate (warm summer) (Csb), characterized by four distinct seasons, a favorable natural environment, relatively uniform precipitation distribution throughout the year, and drier conditions during the summer months. The main crops grown in Phillips County are soybean, corn, cotton, and rice. **Figure 2** shows the phenological calendar of the main crops in U1–U3, which is based on data from the National Agricultural Statistics Service (NASS), United States Department of Agriculture (USDA). The growing seasons of soybean, corn, cotton, and rice are basically between April and September, with soybean and corn having the most overlapping growing seasons and the most similar spectral characteristics (Wei et al., 2023).

The other 9 study areas encompass diverse agricultural regions across North America and Asia, including: (1) seven U.S. counties designated as U4-U10 - Renville County (North Dakota), Butler County (Nebraska), DeWitt County (Illinois), Van Wert County (Ohio), Dodge County (Minnesota), Saline County (Missouri), and Marshall County (Indiana) in the relatively flat central U.S.; (2) two Chinese sites - Nehe City (Heilongjiang Province, C1) in the northeast plains and Lingbi County (Anhui Province, C2) in more mountainous terrain. All regions share similar agricultural timelines with soybean sowing from mid-April to late May and harvest between early September and late November. Primary crops include soybeans, corn, spring wheat, winter wheat, sunflowers, cotton, and rice. Based on farmland regularity, the sites are classified into three categories: regular fields (U1, U2, U7), relatively regular fields (U4-U6, U8), and fragmented fields (U3, U9, U10, C1, C2).

### 2.2 Data

#### 2.2.1 Reference data

The sample data comes from the Cropland Data Layer (CDL) published annually by the National Agricultural Statistics Service (NASS) of the United States Department of Agriculture (USDA) and the ChinaSoyArea10m created by Mei et al. (2024). The CDL data have been generated annually since 1997 for all U.S. states and cover more than 100 crop types (Copenhaver et al., 2021). Since 2008, the CDL has covered the entire continental U.S at a 30-meter resolution (Lin et al., 2022b). The CDL data had 85% to 95% accuracy in classifying major crops (Tran et al., 2022), which is a high-quality reference data in crop mapping studies and widely used in various crop mapping studies (Li et al., 2024; Tran et al., 2022). The ChinaSoyArea10m dataset has achieved good accuracy in comparisons with county and prefecture-level statistical yearbooks and ground sample verification, and can be used as a reference source for soybean sample data in China.

#### 2.2.2 Sentinel-2 data

The Sentinel-2 satellite, developed under the European Space Agency’s Copernicus program, provides multispectral observations across 13 spectral bands covering visible light, near-infrared (NIR), and shortwave infrared (SWIR) bands. Spatial resolution varies by band at 10 meters, 20 meters, and 60 meters, respectively. The Sentinel-2A/2B constellation has a revisit cycle of approximately 5 days (Drusch et al., 2012; Segarra et al., 2020). This study selected 10 bands for analysis (**Table 2**). A significant advantage of Sentinel-2 for agricultural applications is its inclusion of three red edge bands, which are highly sensitive to vegetation chlorophyll content and canopy structure, making them particularly important for crop identification (Clevers and Gitelson, 2013; Qiao et al., 2024). To ensure data continuity, Sentinel-2 data underwent cloud removal and resampling to generate median-synthesized images at 10-day intervals. Missing data in heavily cloudy areas were filled using linear interpolation, and all bands were resampled to 10-meter resolution to maintain consistent spatial resolution. The Sentinel-2 multispectral imagery from 2020 to 2022 used in this study were obtained from the Google Earth Engine (GEE) platform. The 10-day median composite imagery were employed for spectral feature analysis and NSII construction, while single-phase imagery under the “optimal time window” were utilized for NSII validation (**Table 3**).

**Table 2:** Parameters of Sentinel 2 bands used in this study.

Band number	Band name	Central wavelength/nm	Band width/nm	Spatial resolution/m
2	Blue	490	65	10
3	Green	560	35	10
4	Red	665	30	10
5	Red Edge-1 (RE1)	705	15	20
6	Red Edge-2 (RE2)	740	15	20
7	Red Edge-3 (RE3)	783	20	20
8	NIR	842	115	10
8A	Narrow-NIR	865	20	20
11	SWIR1	1610	90	20
12	SWIR2	2190	180	20

**Table 3:** Sentinel-2 data within the optimal time window for each study area.

Sites	Image acquisition date	Number of scenes
	August 19, 2020	4
U1	August 24, 2021	4
	August 4, 2022	4
	August 10, 2020	4
U2	August 23, 2021	4
	August 23, 2022	4
	August 23, 2020	4
U3	September 3, 2021	4
	August 8, 2022	3
	August 24, 2020	4
U4	August 14, 2021	4
	August 14, 2022	4
	August 25, 2020	2
U5	August 5, 2021	2
	August 10, 2022	2
	July 29, 2020	1
U6	September 7, 2021	1
	July 19, 2022	1
	August 27, 2020	3
U7	August 5, 2021	3
	July 21, 2022	3
	August 17, 2020	3
U8	August 4, 2021	3
	August 4, 2022	3
	August 24, 2020	2
U9	August 9, 2021	2
	July 20, 2022	2
	August 20, 2020	2
U10	August 5, 2021	1
	August 18, 2022	2
	August 16, 2020	5
C1	August 31, 2021	5
	August 16, 2022	5
	August 17, 2020	2
C2	August 19, 2021	2
	August 24, 2022	2

## 3 Methods

**Figure 3:**
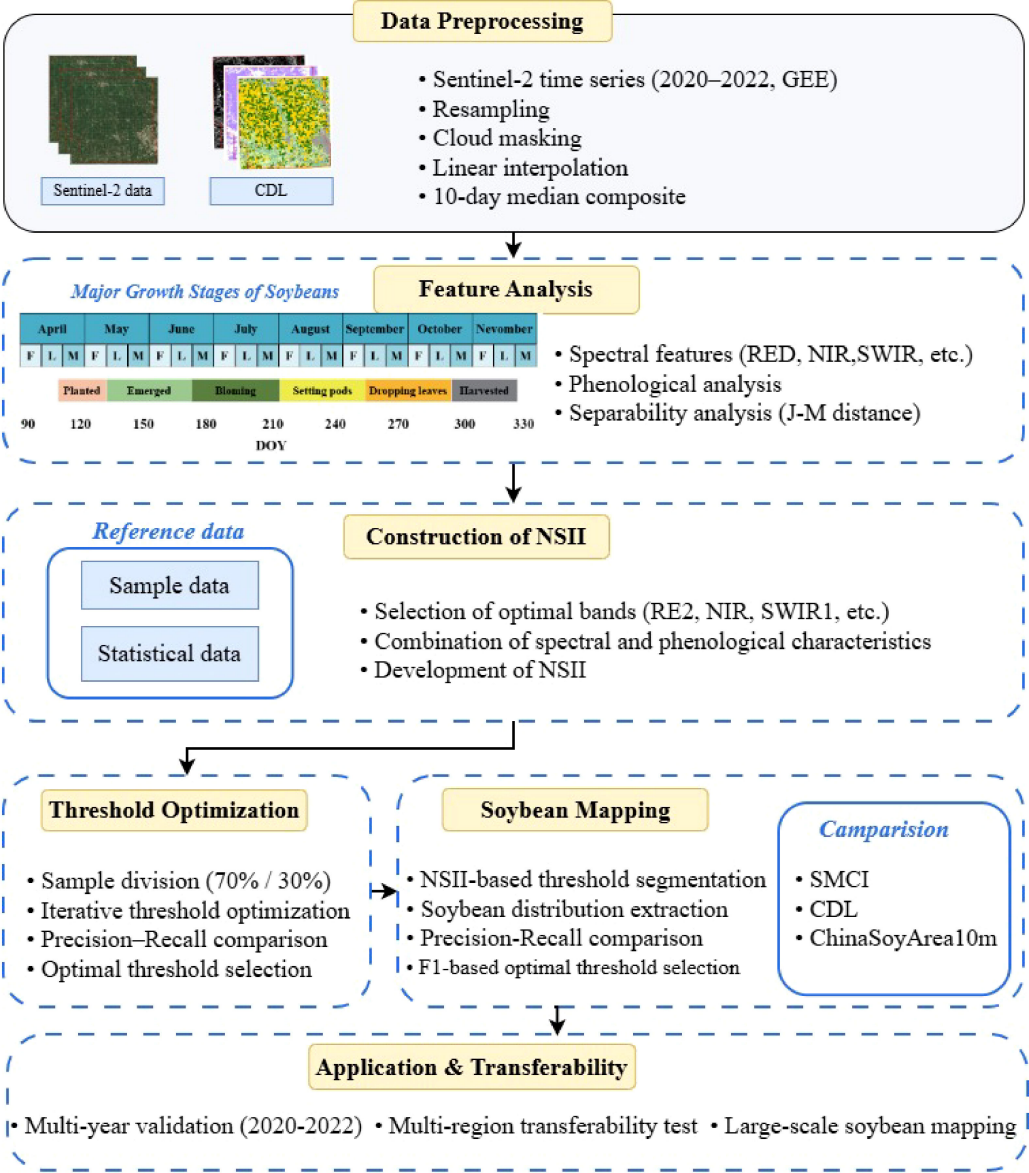
Workflow for soybean mapping.

The workflow of this study comprises four components (**Figure 3**): (1) sample collection from different crops; (2) phenological analysis and spectral dynamics analysis; (3) determination of temporal windows and construction of the Novel Soybean Identification Index (NSII); (4) spatiotemporal transfer validation of NSII and evaluation of mapping accuracy.

### 3.1 Crop type sample data acquisition

The sample data consists of two parts: one part is used to develop a new soybean identification index, referred to as the “reference sample”; the other part is used to verify the accuracy of the soybean spatial distribution map based on this index, referred to as the “test sample”. All samples are uniformly distributed throughout the entire study area. The reference samples are exclusively sourced from the U1-U3 regions, obtained as follows: First, the agricultural land areas with annual cloud cover below 15% in 2021 are cropped out. Then, pixels with crop type confidence levels exceeding 95% are extracted from the CDL crop layer within this area. The test samples were categorized into two types: soybeans and non-soybeans. The acquisition method was similar to that of the reference samples, but after extracting pixels with crop type confidence levels exceeding 95%, a portion of random sampling points were further selected through random sampling. To reduce spatial autocorrelation between samples, the minimum distance between sample points was set to exceed 30 meters (i.e., 3 pixels). The final number of samples obtained for each study area is shown in **Table 4**.

**Table 4:** Sample information.

Sample type	Location	Sample size	
		Soybean	Non-soybean
Reference sample	U1	416060	408882
	U2	476971	1017095
	U3	386471	346313
Test sample	U1	9791	11269
	U2	21831	31355
	U3	14504	9767
	U4	24411	52123
	U5	11425	16398
	U6	11017	11327
	U7	13581	10807
	U8	7875	12482
	U9	17524	19585
	U10	5421	10368
	C1	14972	14518
	C2	14740	12352

### 3.2 Time-series feature curves and separability analysis of crops

Based on the preprocessed Sentinel-2 data, 35 vegetation indices were further calculated (**Table 5**), and the feature curves of 10 spectral bands and the 35 vegetation indices at 10-day intervals during the soybean growing season were finally obtained. In addition, based on the reference sample obtained in section 3.1 Crop type sample data acquisition, we evaluated the separability between soybean and corn across features (May–October) using the Jeffries-Matusita (J-M) distance (Qiu et al., 2014). The J-M distance can be effective in evaluating the separability between different crop types (Wang et al., 2018) (Equations 1–3). By analyzing temporal spectral feature curves and crop separability metrics, the optimal time window and features for soybean identification were determined.

**Table 5:** Vegetation indices used in this study.

Spectral index	Formula	References
Normalized Difference Vegetation Index (NDVI)	NDVI=(B8−B4)/(B8+B4)	(Rouse et al., 1974)
Normalized Difference red-edge 1 (NDre1)	NDre1=(B6−B5)/(B6+B5)	(Fernández-Manso et al., 2016)
Normalized Difference red-edge 2 (NDre2)	NDre2=(B7−B5)/(B7+B5)	(Fernández-Manso et al., 2016)
Land Surface Water Index (LSWI)	LSWI=(B8−B11)/(B8+B11)	(Xiao et al., 2005)
Soil Adjusted Vegetation Index (SAVI)	SAVI=1.5×(B8−B4)/(B8+B4+0.5)	(Huete, 1988)
Normalized Difference Water Index (NDWI)	NDWI=(B3−B8)/(B3+B8)	(McFeeters, 1996)
Plant Senescence Reflectance Index (PSRI)	PSRI=(B4−B3)/B6	(Fernández-Manso et al., 2016)
Normalized Difference Vegetation Index red-edge 1 (NDVIre1)	${\text{NDVI}}_{\text{re}}1=(\text{B}8-\text{B}5)/(\text{B}8+\text{B}5)$	(Gitelson and Merzlyak, 1997)
Normalized Difference Vegetation Index red-edge 2 (NDVIre2)	${\text{NDVI}}_{\text{re}}2=(\text{B}8-\text{B}6)/(\text{B}8+\text{B}6)$	(Gitelson and Merzlyak, 1997)
Novel inverted red-edge chlorophyll index (IRECI)	IRECI=(B8−B4)/(B5/B6)	(Zhang et al., 2022a)
Chlorophyll Index red-edge (Cire)	CIre=(B8/B5)−1	(Zhang et al., 2022a)
Ratio Vegetation Index (RVI)	RVI=B8/B4	(Jordan, 1969)
Wide Dynamic Range Vegetation Index (WDRVI)	WDRVI=(a×B8−B4)/(×B8−B4), a=0.1	(Gitelson, 2004)
Non-Linear vegetation Index. (NLI)	NLI=(B8×B8−B4)/(B8×B8+B4)	(Goel and Qin, 1994)
Modified Non-linear vegetation Index (MNLI)	MNLI=[(B8×B8−B4)×(1+0.5)]/(B8×B8+B4+0.5)	(Gong et al., 2003)
Optimization of Soil-Adjusted Vegetation Indices (OSAVI)	OSAVI=(B8−B4)/(B8+B4+0.16)	(Rondeaux et al., 1996)
Enhanced Vegetation Index (EVI)	EVI=2.5×(B8−B4)/(B8+6×B4−7.5×B2+1)	(Huete et al., 2002)
Difference Vegetation Index (DVI)	DVI=B8−B4	(Fernández-Manso et al., 2016)
Bareness soil index (BSI)	BSI=[(B11+B4)−(B8+B2)]/[(B11+B4)+(B8+B2)]	(Bera et al., 2020)
Winter Rapeseed Index (WRI)	WRI=[(B8−B3)/B8+B3]×[B2/(B3+B4)]	(Zhang et al., 2022b)
Ratio Vegetation Index Green (RVI_Green)_	${\text{RVI}}_{\text{Green}}=\text{B}8/\text{B}3$	(Gitelson et al., 1996)
Red light and red edge band ratio vegetation index (SR_Red/Green_)	${\text{SR}}_{\text{Red/Green}}=\text{B}4/\text{B}3$	(Chappelle et al., 1992)
Visible Atmospherically Resistant Index (VARI_Green_)	${\text{VARI}}_{\text{Green}}=(\text{B}3-\text{B}4)/(\text{B}3+\text{B}4-\text{B}2)$	(Gitelson et al., 2002)
Triangular Vegetation Index (TVI)	TVI=a×(b×(B8−B3)−c×(B4−B3)) a=0.5,b=120,c=200	(Haboudane et al., 2004)
MERIS Terrestrial Chlorophyll Index (MTCI)	MTCI=(B6−B5)/(B5−B4)	(Dash and Curran, 2007)
Modified Normalized Difference Vegetation Index (MNDVI)	MNDVI=(B3−B11)/(B3+B11)	(Dihkan et al., 2013)
Modified Simple Ratio Vegetation Index (MSAVI)	$\text{MSAVI}=[2\times \text{B}8+1-\sqrt{{\left(2\times \text{B}8+1\right)}^{2}-8\times (\text{B}8-\text{B}4)}]/2$	(Haboudane et al., 2004)
Modified Simple Ratio red-edge (MSRre)	$\text{MSRre}=[(\text{B}8/\text{B}5)-1]/[\sqrt{(\text{B}8/\text{B}5)-1}]$	(Fernández-Manso et al., 2016)
Modified Simple Ratio red-edge Narrow (MSRren)	$\text{MSRren}=[(\text{B}8\text{A}/\text{B}5)-1]/[\sqrt{(\text{B}8\text{A}/\text{B}5)-1}]$	(Fernández-Manso et al., 2016)
Renormalized Difference Vegetation Index (RDVI)	$\text{RDVI}=(\text{B}8-\text{B}4)/(\sqrt{\text{B}8+\text{B}4})$	(Haboudane et al., 2004)
Simple Ratio (SR)	SR=B8/B4	(Fernández-Manso et al., 2016)
Red-Edge Position (REP)	REP=705+35×[0.5×(B4+B7)−B5]/(B6−B5)	(Guyot et al., 1988)
Normalized Burn Ratio (NBR)	NBR=(B8−B12)/(B8+B12)	(Fernández-Manso et al., 2016)
Greenness and Water Content Composite Index (GWCCI)	GWCCI=B11×(B8−B4)/(B8+B4)	(Chen et al., 2023)
Soybean Mapping Composite Index (SMCI)	$\text{SMCI}={\text{TW}}_{\text{Global}}\{(\text{B}11+\text{B}8+\text{B}8\text{A}+\text{B}7+\text{B}6)\times \text{EVI}\times \text{GCVI}\}$ EVI=2.5×(B8−B4)/(B8+6×B4−7.5×B2+1), GCVI=(B8/B3)−1 ${\text{TW}}_{\text{Global}}\{\;\;\}$ is the time period	(Xiao et al., 2024)

*Bi* denotes the *i*th band of Sentinel-2.

(1) $$\text{JM}(j,k)=\int_{\text{x}}{\left[\sqrt{P(x∥j)}-\sqrt{P(x∥k)}\right]}^{2}\text{d}x$$

where *j* and *k* denote two different crop types; *x* is the value for the different features. P(x∥j) and P(x∥k) are probability density functions (PDFs) of feature *x* for classes *j* and *k*, and Equation 1 can be simplified to Equation 2

(2) $$\text{JM}=2(1-{\text{e}}^{-\text{B}})$$

(3) $$\text{B}=\frac{1}{8}\sqrt{{\left(\mu_{j}-{\text{μ}}_{\text{k}}\right)}^{\text{T}}{\left[(\sum j+\sum k)/2\right]}^{-1}(\mu_{j}-\mu_{k})}+\frac{1}{2}\ln[(∥(\sum j+\sum k)/2∥)/(\sqrt{∥\sum j∥∥\sum k∥})]$$

Where *μ_j_* and *μ_k_* represent the average spectral reflectance of a single crop category, Σ*j* and Σ*k* are unbiased estimates of the covariance matrices of specific classes *j* and *k*. The J-M distance takes values in the range of 0 to 2. The greater the feature difference between the two crops, the greater the value of the J-M distance (Gxokwe et al., 2022).

**Figure 4:**
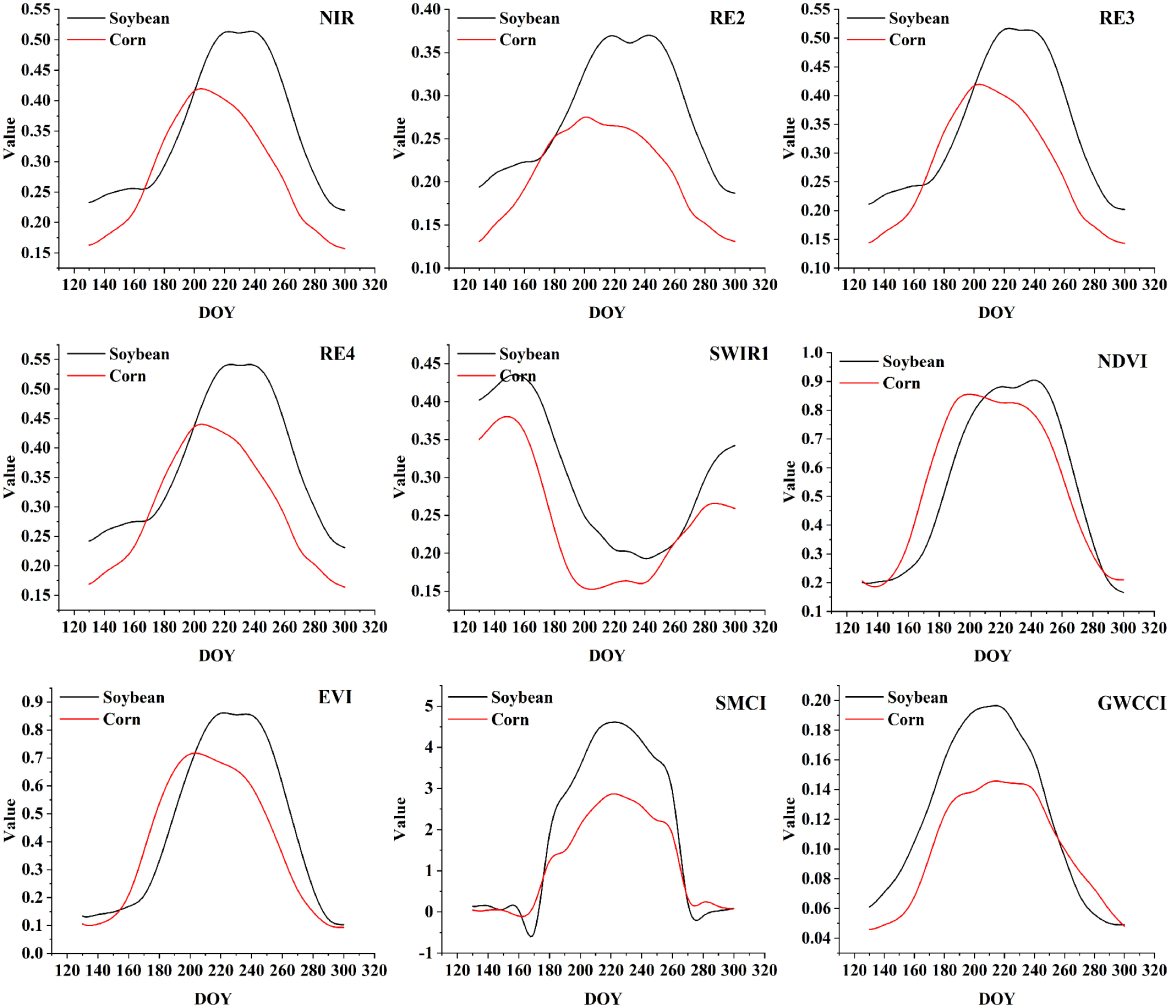
Some examples of feature curves.

**Figure 5:**
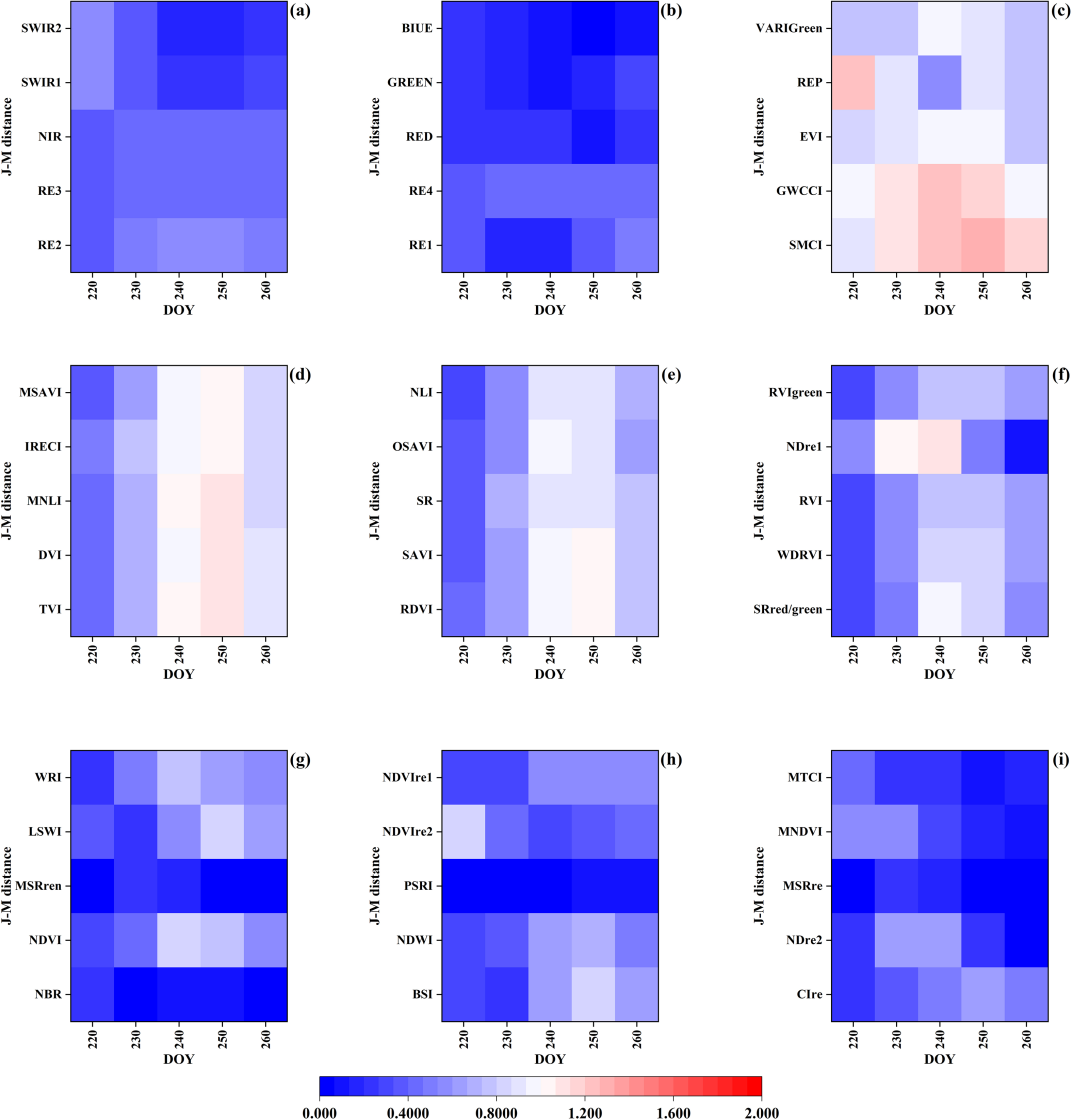
J-M distance calculated based on different features.

Due to space constraints, the complete set of feature curves for different crops across 12 study regions and their corresponding separability analysis matrices are not fully presented. Among crops planted during the same period, corn exhibits the highest spectral confusion with soybean. Using U1 as a representative case, we present the temporal curves of some typical features for corn and soybean (**Figure 4** and **Supplementary Material**) and the separability heatmap of different features (**Figure 5**). The performance of the same feature varies in different regions, but overall, there are significant and stable feature differences between soybeans and corn from early August (DOY ≈ 220) to mid-September (DOY ≈ 260). During this time window, soybeans are primarily in the pod-filling stage, while corn progresses from silking to milk stage. In this phase, soybeans exhibit distinctive characteristics: rapid seed enlargement within pods, initial yellowing and shedding of lower leaves, significant reduction in moisture content, cessation of plant height growth, and increased canopy openness, leading to sharp declines in red-edge band reflectance and marked increases in short-wave infrared (SWIR) band reflectance. Conversely, corn reaches peak leaf area index (LAI) during silking while maintaining consistently high near-infrared (NIR) reflectance; upon entering the milk stage, basal leaves begin to yellow, red-edge indices show gradual decline, and SWIR reflectance remains relatively stable. These pronounced phenological differences and spectral characteristic variations provide critical discriminative basis for simultaneous crop classification during this growth period. Therefore, we identify the period from August to mid-September as the optimal temporal window for soybean identification. During the optimal temporal window, the spectral bands with high separability include the red-edge bands (RE2, RE3, RE4), NIR bands, and SWIR bands (**Figure 5**). Among them, RE2 has the highest average separation degree (average J-M=0.50). The vegetation indices with high separability include the SMCI (average J-M=1.12), GWCCI (average J-M=1.08), and EVI (average J-M=0.88). Among them, SMCI and GWCCI are indices developed specifically for soybean identification. These bands and indices can be used as the basic features and references for the development of NSII in this study.

### 3.3 Principles and methods of novel index construction

**Figure 5** shows that the red-edge bands (RE2, RE3, RE4), the NIR bands and the SWIR bands, SMCI, GWCCI, and EVI have a better ability to distinguish between soybeans and corn. RE2 performs best in the three red-edge bands; SWIR1 performs better in the two SWIR bands. SMCI uses eight bands for calculation and takes EVI into account. GWCCI is the product of SWIR1 and NDVI, using red, NIR, and SWIR bands for calculation. In order to comprehensively use different spectral information and reduce feature redundancy, we selected RE2 from three red-edge bands, SWIR1 from two SWIR bands, and EVI from vegetation indices to construct NSII. EVI can provide supplementary information of red and NIR bands.

**Figure 6:**
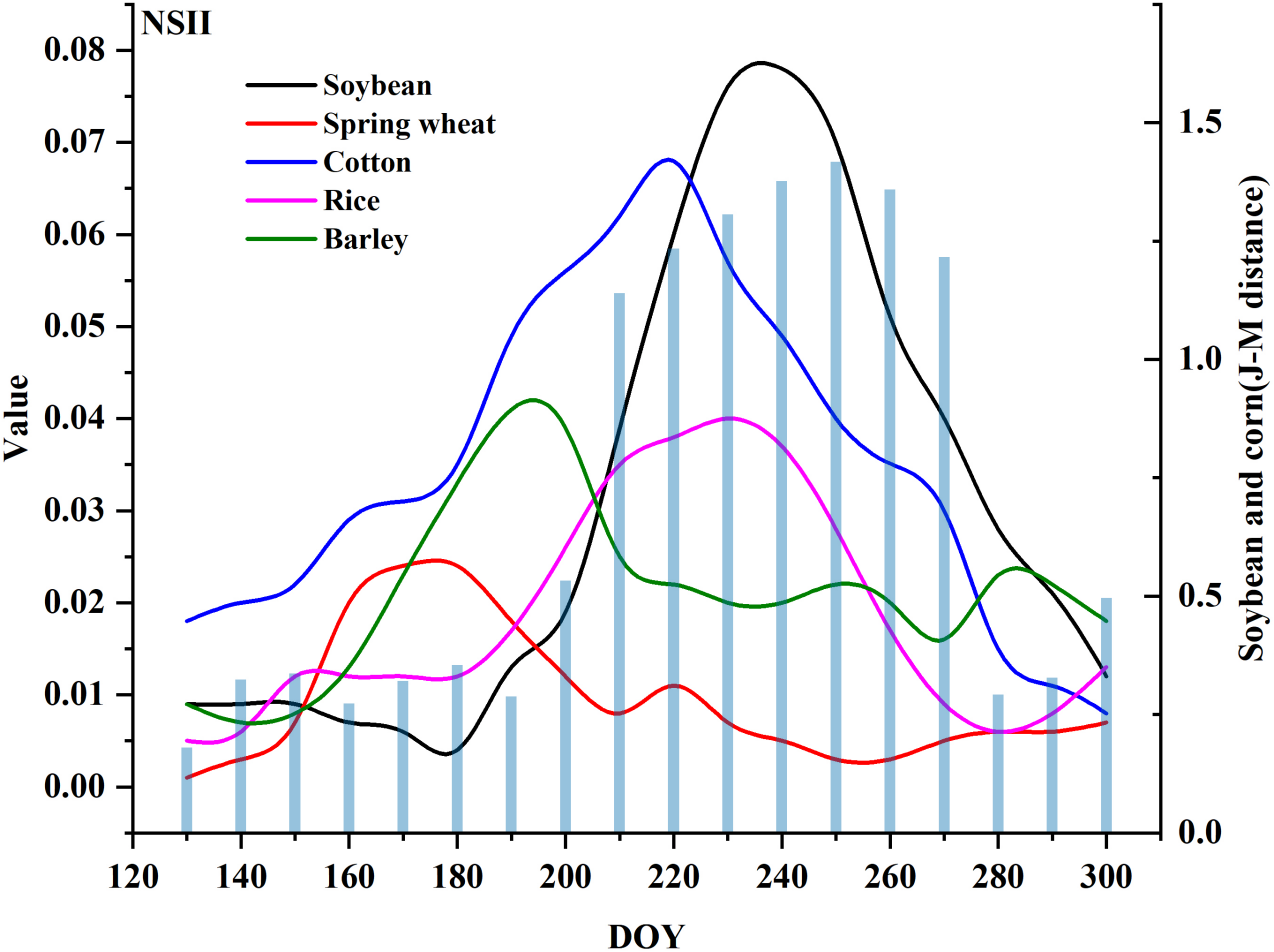
Spectral curves of soybeans and their corresponding crops and the separability of soybeans and corn (J-M distance).

By performing mathematical processing on the three selected features (such as addition, subtraction, multiplication, division, and reciprocal transformation) to enhance the spectral feature information of soybeans, and selecting the mathematical combination with the highest average J-M distance under the optimal time window as the calculation formula for NSII (Equations 4, 5). The higher the NSII value, the higher the likelihood that the pixel is soybean. Comparative analysis of **Figure 5** and **6** demonstrates that the NSII index developed in this study significantly outperforms existing indices in terms of J-M distance between soybean and corn. Moreover, this index consistently maintains an average separability above 1.05 for distinguishing soybean from other minor crops, such as barley, oats, cotton, Spring wheat, sunflower.

(4) $$\text{NSII}=\sqrt[{3}]{\text{RE}2\times \text{SWIR}1\times \text{EVI}}$$

(5) $$\begin{array}{c} \text{EVI}=\text{G}\times (\text{NIR}-\text{Red})/(\text{NIR}+{\text{C}}_{1}\times \text{Red}-{\text{C}}_{2}\times \text{Blue}+\text{L}),\\ \text{G}=2.5,{\text{C}}_{1}=6,{\text{C}}_{2}=7.5,\text{L}=1 \end{array}$$

where RE2, SWIR1, Red, NIR, and Blue correspond to the B6, B11, B4, B8, and B2 bands of Sentinel 2, respectively.

### 3.4 Validation and application of NSII

To assess the effectiveness of NSII, we conducted a three-year (2020–2022) soybean mapping experiment at 12 locations in major soybean-producing regions in the United States and China, and compared the results with CDL and the ChinaSoyArea10m dataset. In addition, considering that SMCI has the highest separability among existing indices (**Figure 5**), we further compared the results of NSII-based and SMCI-based soybean mapping. Specifically, the soybean planting areas were extracted by the automatic thresholding method. The steps for threshold determination are as follows:

1. Sample division: The “test sample” in each region is randomly split into two parts in a 7:3 ratio, with 70% used for threshold determination and 30% for accuracy verification.

2. Initial threshold range determination: calculate the minimum value ( ${\text{T}}_{\text{min}}$) and maximum value ( ${\text{T}}_{\text{max}}$) of NSII.

3. Threshold Calculation:

- Set the initial threshold ${\text{T}}_{1}=({\text{T}}_{\text{min}}+{\text{T}}_{\text{max}})/2$ and split the image into two parts.
- Compute four classification metrics: Overall Accuracy(OA), Precision (P), Recall (R), and F1 score. OA measures the percentage of correctly classified pixels. Precision represents the fraction of predicted soybean pixels that were actual soybeans, while Recall indicates the proportion of actual soybean pixels correctly identified. The F1 score, as the harmonic mean of Precision and Recall, provides a balanced performance assessment.
- Compare P and R: if P>R (indicating the commission errors are larger than the omission errors and the threshold is set too low), update the threshold as ${\text{T}}_{2}=({\text{T}}_{1}+{\text{T}}_{\text{max}})/2$; if P<R (indicating the omission errors are larger than the commission errors and the threshold is set too high), update the threshold as ${\text{T}}_{2}=({\text{T}}_{\text{min}}+{\text{T}}_{1})/2$.

4. Iterative Optimization: Compare the F1 scores before and after updating the threshold. If the F1 score improves, continue iterating using the same logic.

5. Final Threshold and Verification: Repeat the process until the optimal threshold is obtained. Due to regional variability, the optimal thresholds differ across regions. Verify the accuracy using the remaining 30% of the samples.

## 4 Results

### 4.1 Soybean mapping results and accuracy evaluation

**Figure 7:**
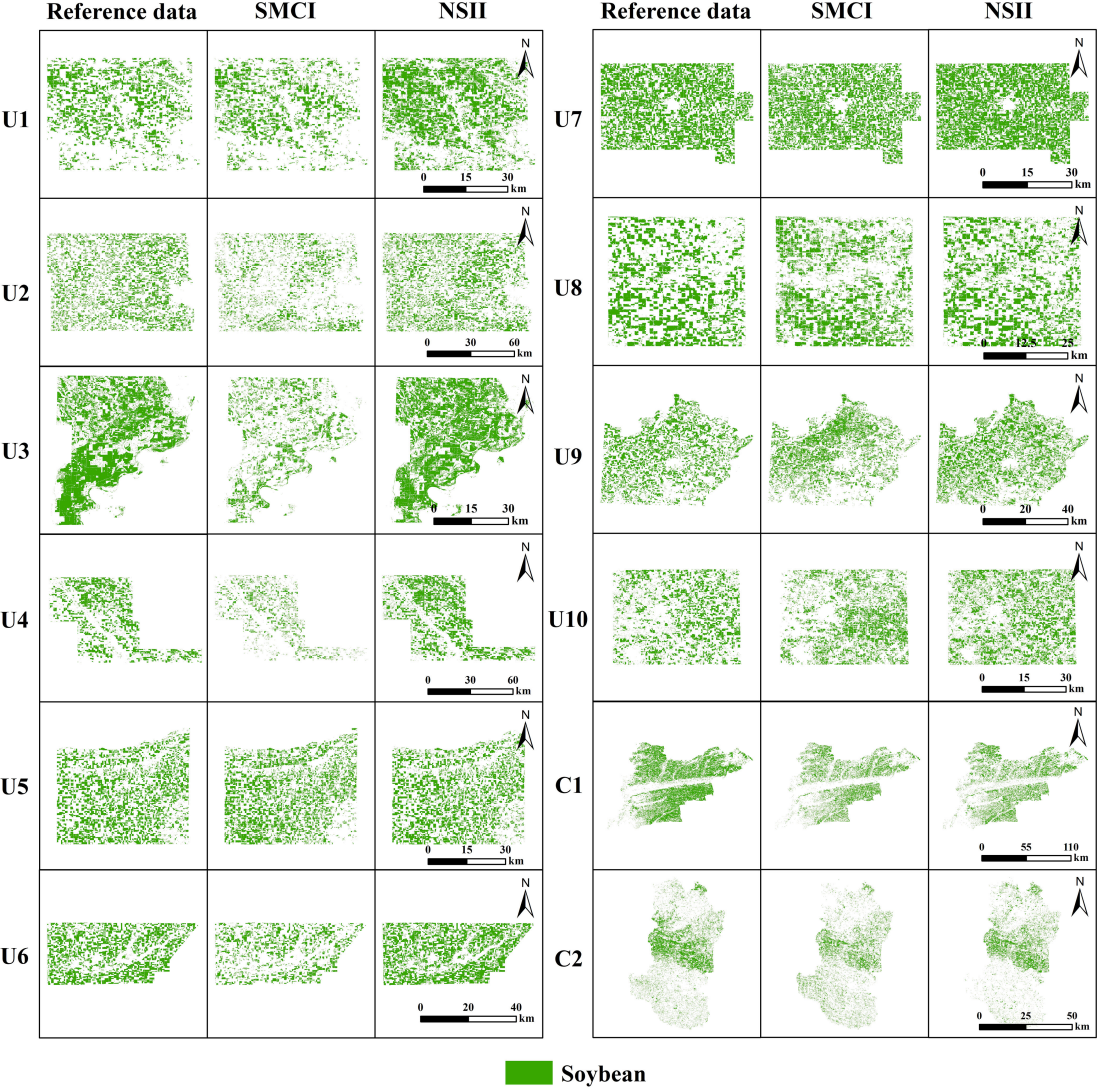
Reference, SMCI-based and NSII-based soybean distribution maps in 12 study areas in 2021.

**Figure 8:**
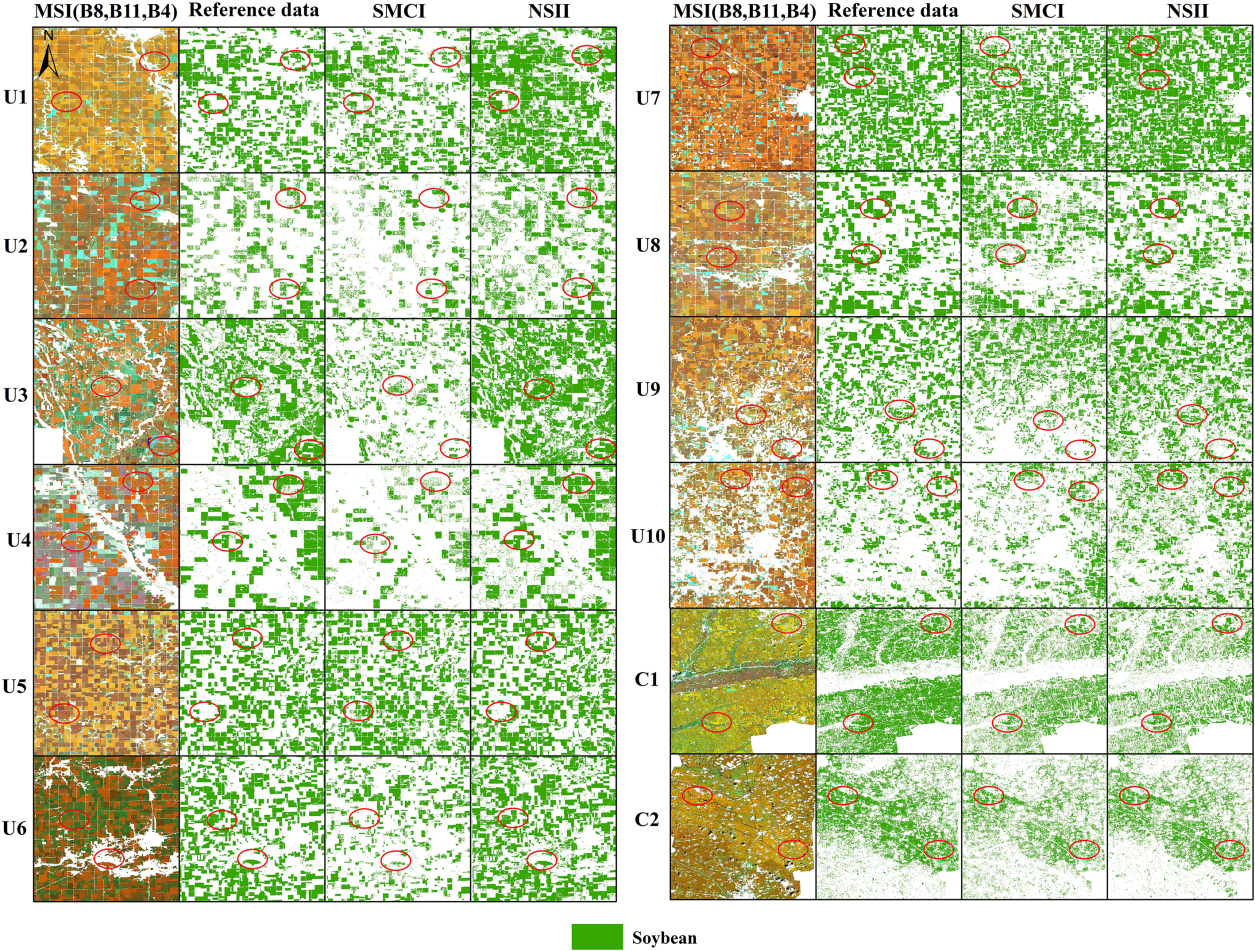
Zoom images of MSI (composite multispectral image of band8, 11 and 4), Reference dataset, SMCI-based, and NSII-based soybean distribution maps.

**Figure 7** shows the reference soybean distribution in 12 study areas and the mapped results based on NSII and SMCI methods, while **Figure 8** provides detailed views of representative local areas from **Figure 7**. Overall, compared to the SMCI-based soybean distribution maps, the NSII-based maps showed higher consistency with the CDL data in both regular (e.g., U1, U2, U7) and fragmented (e.g., U3, U9) study areas. The NSII results for regions C1 and C2 are also closer to the ChinaSoyArea10m dataset. In SMCI-based soybean distribution maps, there were significant omissions in U2, U3, U4, U6 and commission in U5. In contrast, the NSII method demonstrated significantly improved accuracy in these corresponding study areas, markedly reducing both omission and commission errors of soybean pixel classification (as shown by the red circles in **Figure 8**).

The soybean mapping accuracy derived from the NSII was systematically quantified and comparatively analyzed against the SMCI method (**Table 6**). The average precision, recall, F1 score, and accuracy of soybean mapping results based on SMCI in 12 regions are 0.79, 0.71, 0.74, and 0.77, respectively, while the average precision, recall, F1 score, and accuracy of soybean mapping results based on NSII are 0.79, 0.83, 0.80, and 0.85, respectively. Precision remained unchanged, while recall, F1 score, and accuracy increased by 12, 6, and 8 percentage points, respectively. This means that the soybean mapping results based on NSII have fewer omission errors. Overall, NSII outperformed SMCI in all regions, providing a better feature index for accurate mapping of soybean.

**Table 6:** Accuracy comparison of NSII and SMCI at different sites.

Sites	Methods	Precision	Recall	F1 score	Accuracy
U1	NSII	0.72	0.86	0.78	0.84
	SMCI	0.74	0.73	0.73	0.78
U2	NSII	0.71	0.85	0.77	0.83
	SMCI	0.74	0.70	0.72	0.75
U3	NSII	0.75	0.85	0.80	0.85
	SMCI	0.78	0.67	0.72	0.7
U4	NSII	0.74	0.87	0.80	0.89
	SMCI	0.78	0.72	0.75	0.79
U5	NSII	0.80	0.85	0.82	0.84
	SMCI	0.76	0.76	0.76	0.8
U6	NSII	0.75	0.92	0.83	0.91
	SMCI	0.80	0.75	0.77	0.81
U7	NSII	0.81	0.89	0.85	0.92
	SMCI	0.84	0.80	0.82	0.82
U8	NSII	0.81	0.84	0.82	0.83
	SMCI	0.74	0.73	0.73	0.79
U9	NSII	0.79	0.88	0.83	0.85
	SMCI	0.76	0.79	0.77	0.78
U10	NSII	0.83	0.90	0.86	0.89
	SMCI	0.79	0.81	0.80	0.83
C1	NSII	0.94	0.61	0.73	0.78
	SMCI	0.91	0.56	0.69	0.75
C2	NSII	0.94	0.67	0.78	0.80
	SMCI	0.89	0.52	0.65	0.70

### 4.2 Evaluation of NSII’s multi-year mapping results and transferability

**Figure 9:**
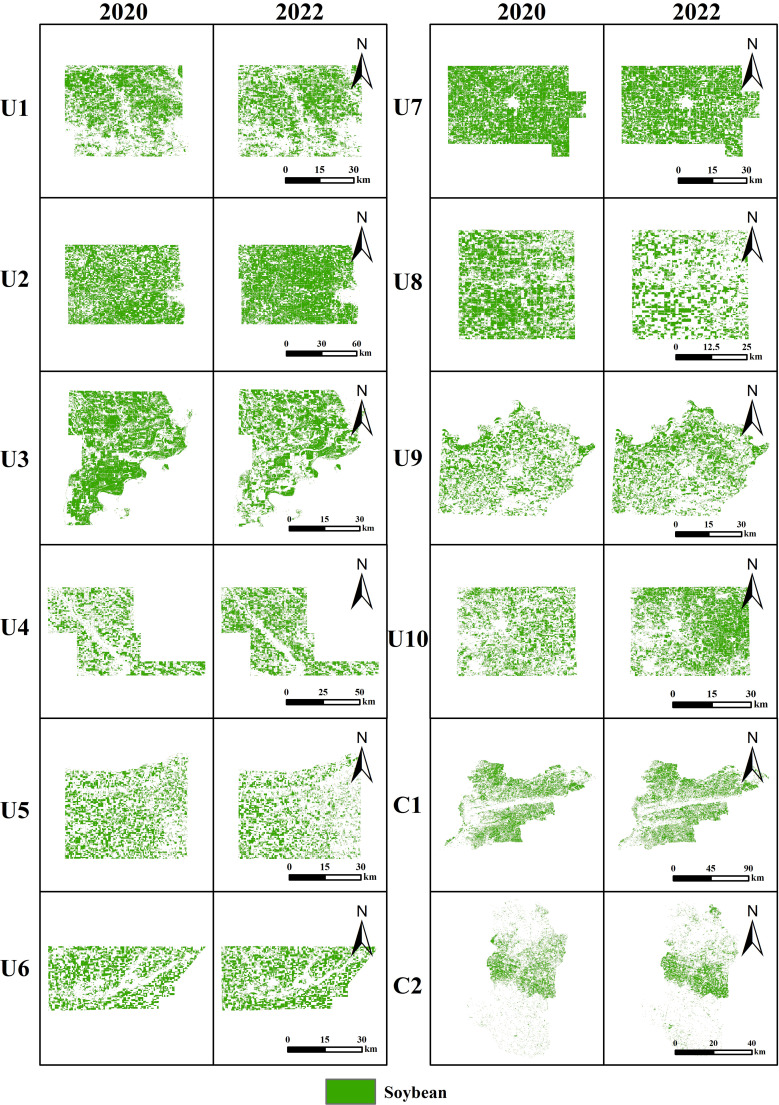
Soybean distribution maps based on NSII in 2020 and 2022.

**Figure 10:**
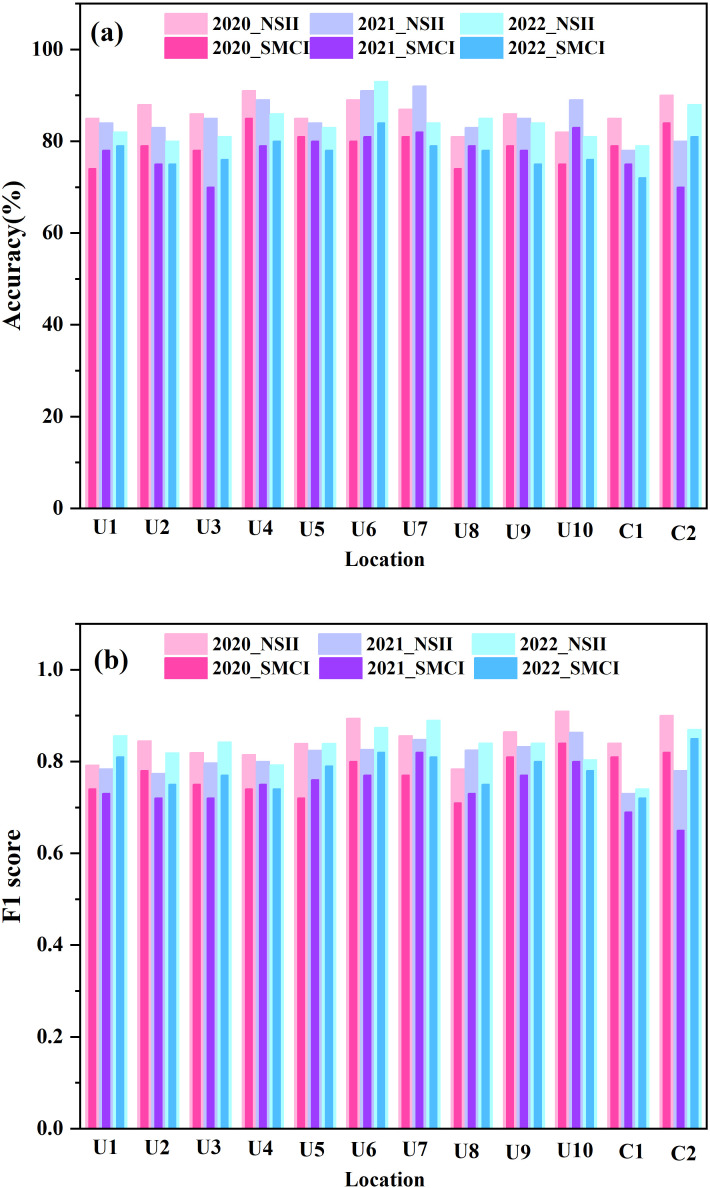
Accuracy and F1 score of soybean field distribution in each study area from 2020 to 2022.

In order to better evaluate the performance of NSII over multiple years, distribution mapping was conducted in 12 study areas for three consecutive years. **Figure 9** shows the distribution of soybeans in all regions in 2020 and 2022, and **Figure 10** shows the soybean field distribution accuracy and F1 score in each study area from 2020 to 2022.

Comparing **Figure 7** and **Figure 9**, it is found that there are obvious changes in the distribution and area of soybeans in some areas. For example, from 2020 to 2022, the soybean planting area of U4 and U10 gradually increased, while the soybean planting area of U1 showed a downward trend within three years. These changes are consistent with the trends of CDL and soybean planting area statistics in the USDA QuickStats database. The soybean planting area of U6 and U9 has remained basically unchanged from 2020 to 2022. The soybean cultivated area in C1 exhibited a concave trend (decrease in 2021and recovery in 2022), whereas C2 maintained stable spatial distribution patterns (interannual variation). The observed soybean area dynamics in regions C1 and C2 show strong consistency with the official statistics from China Statistical Yearbook. Overall, the 2020–2022 mapping results demonstrated logical consistency across all study regions.

From **Figure 10**, it can be seen that the accuracy and F1 score of each study area changed very little among three years. Specifically, the average F1 score for all regions from 2020 to 2022 was 0.82, with an average standard deviation of 0.03; the average accuracy for all regions from 2020 to 2022 was 0.85, with an average standard deviation of 0.03. This fully demonstrates the excellent stability of NSII over the years.

## 5 Discussion

Among all the Sentinel-2 bands involved in the calculation of NSII, the separability of RE2 was the best, and the other red-edge bands also showed high separability, which indicated that the red-edge bands were very effective in distinguishing soybeans and corn, which was in line with the previous studies (Feng et al., 2019). The red edge band is located between the visible and NIR bands, corresponding to the transition region where the spectral reflectance of vegetation increases sharply. It is highly sensitive to the chlorophyll content, leaf structure and biomass of vegetation, and is a key indicator band for the physiological state of vegetation. The red-edge band is less reflective of non-vegetation backgrounds such as soil, which helps to minimize the effect of mixed pixels and highlights the canopy information of soybean. The NIR and SWIR1 bands are also important for identifying soybeans, which is consistent with the conclusions of Yin et al. (2020) and She et al. (2020). In addition to the original bands, some remote sensing indices are better than the spectral bands for identifying soybeans, such as SMCI, GWCCI, EVI and REP. These indices are mostly correlated with RE, NIR, and SWIR bands. This further proves the effectiveness of these bands in soybean mapping. There is not much difference between soybeans and corn in the early stage of growth, while corn is significantly higher than soybeans in the middle stage of growth. When the transpiration rate of corn is faster than that of soybeans, more water will be stored in the leaves and canopy, resulting in a smaller SWIR value. In addition, the difference in canopy greenness between soybean and corn is more pronounced during the mid-growth stage (Xiao et al., 2024). In this study, we combined the separability analysis results with the unique biophysical characteristics of soybean in the middle of the growing season to design NSII, a remote sensing identification feature for the middle of the growing season of soybean.

The mid-season stage of soybean growth (from planting to pod set) is a critical period for determining yield and quality (Zeleke and Nendel, 2024). This stage requires careful management of fertilization, irrigation and pest control to ensure plant health and soybean pod formation. Real-time monitoring of soybeans can help optimize resource allocation and improve the efficiency and sustainability of agricultural production. However, the spectral overlap between soybean and other major crops, such as corn, poses a significant challenge for timely and accurate soybean mapping (Lin et al., 2022a; Zhang et al., 2020). Traditional remote sensing indices, such as NDVI, are often ineffective for crop identification during the peak growing season. This is because high levels of crop greenness, water content, and other biophysical parameters lead to NDVI saturation (Mutanga et al., 2023). To address this limitation, researchers have developed novel spectral indices to improve the discrimination of soybean from other co-occurring crops and to provide more precise decision-making support for agricultural management. While significant progress has been made, existing methods still have room for improvement. For example, the GWCCI proposed by Chen et al. (2023) underutilizes the red-edge bands, which are critical for capturing subtle differences in crop characteristics. The SMCI proposed by Xiao et al. (2024) employs eight spectral bands, resulting in computational complexity and redundant band information. NSII is proposed in this study to overcome above limitations and it offers the following advantages:

1. Simple calculation process. It adopts a simple and easy-to-understand calculation process. By screening spectral bands and remote sensing indices, it efficiently combines bands and indices with higher J-M distances for soybean identification. This reduces computational complexity, saves time and computational resources for data processing, and thereby improves the overall efficiency of mapping.
2. Good soybean identification capability. The analysis of J-M distance shows that the separability of NSII > SMCI > GWCCI (**Figure 5** and **Figure 6**). Additionally, the soybean identification accuracy based on NSII is higher than that based on SMCI.
3. High stability and transferability. The NSII index has been tested and applied over three years in multiple regions with different planting structures and climatic conditions, consistently achieving high accuracy (Accuracy>80%). This demonstrates its strong spatiotemporal transferability.
4. Potential for mid-season mapping. NSII does not require data from the entire growing season. It can generate soybean distribution maps using data from the optimal time window, which is relatively long. This facilitates the acquisition of high-quality data and enhances the practical applicability of NSII.

The focus of this study is on developing and validating NSII, therefore only a simple threshold method was used for testing in soybean classification. Unlike supervised classification methods, threshold methods only require a single feature band. However, NSII can be combined with other indices to use supervised classifiers for single target crop (such as soybean) or multi-target crop (such as soybean and corn) classification, in order to improve the overall accuracy of crop mapping.

In recent years, Sentinel-2 imagery has been widely applied to soybean and winter crop classification due to its high spatial resolution and advantages in the red edge band (Sabljić et al., 2024). Existing studies indicate that Sentinel-2 data, when combined with machine learning classifiers such as random forests and support vector machines, demonstrate excellent performance in soybean mapping and achieve high classification accuracy across diverse agroecological regions (Feng et al., 2019; Wei et al., 2023). Similarly, Sentinel-2 imagery has been successfully applied to winter crop identification (Bajić et al., 2022), particularly by leveraging the red edge and shortwave infrared bands to capture phenological differences (Liu et al., 2021; Sun et al., 2020). These studies highlight the importance of spectral feature selection and phenological timing in crop discrimination.

Although NSII has significant advantages over traditional soybean mapping, there are still some limitations. Firstly, due to factors such as variety, climate conditions, and planting structure, there are differences in the optimal time window required for different needs, and it is necessary to determine the optimal time window for the target area before calculation. To facilitate the application of NSII in new regions, this paper provides supplementary guidance on determining the optimal time window. Based on the findings of this study, the optimal time window typically corresponds to the mid-growth stage of soybeans, specifically the transition from vegetative growth to pod-setting. At this stage, soybean canopy structure, chlorophyll content, and moisture status exhibit significant differences from corn. In temperate regions, this phase generally occurs between days 180–240, though the exact timing may vary depending on local climate and planting dates. When applying NSII to new regions, users are advised to first analyze temporal vegetation indices (e.g., spectral bands, NDVI, EVI, and other remote sensing indices) to identify the rapid growth phase and peak canopy development period. Subsequently, separability analysis between soybeans and major companion crops (e.g., J-M distance) can be conducted within this phenological window to identify the optimal time range. This phenology-guided approach enhances the transferability and robustness of NSII across diverse agroecological regions. Secondly, in regions with frequent cloud cover, such as tropical or monsoon climates, the applicability of NSII may be constrained. During optimal observation windows, high cloud cover significantly reduces the availability of high-quality optical imagery. While 10-day synthesis and interpolation can partially compensate for observation gaps, persistent cloud contamination still compromises the reliability of spectral features and mapping accuracy. To overcome this limitation, future research could explore multi-sensor fusion strategies, such as combining Sentinel-2 optical data with cloud-penetrating Sentinel-1 SAR imagery or integrating Landsat data. Data fusion approaches could further enhance NSII’s robustness and applicability in heavily cloud-covered regions.

## 6 Conclusions

This study developed a novel soybean identification index (NSII) to address the challenges of spectral overlap. By integrating the RE2, SWIR1, and EVI features, NSII significantly improves the separability of soybean from other crops, particularly corn, within the optimal time window. Results from soybean fields in 12 regions between 2020 and 2022 showed that NSII had an average accuracy of 0.85 and an average F1 score of 0.80, which were 8 and 6 percentage points higher than those of SMCI, respectively. NSII’s computationally efficient design reduces computational complexity, while its stability and transferability across diverse climatic and cropping conditions highlight its practical applicability for large-scale soybean mapping. Furthermore, NSII’s ability to operate within a relatively long optimal time window enhances its feasibility for real-time monitoring and mid-season mapping. These advantages make NSII a valuable tool for precision agriculture, enabling efficient resource allocation, improved crop management, and enhanced food security. Future research could explore the integration of NSII with machine learning models or its application in other regions with complex cropping systems to further validate its robustness and scalability.

## Data Availability

The original contributions presented in the study are included in the article/**Supplementary Material**. Further inquiries can be directed to the corresponding authors.
